# Antibiotic Utilization in Hospitalized Children with Bronchiolitis: A Prospective Study Investigating Clinical and Epidemiological Characteristics at a Secondary Hospital in Madrid (2004–2022)

**DOI:** 10.3390/pathogens12121397

**Published:** 2023-11-28

**Authors:** María Luz García-García, Sonia Alcolea, Patricia Alonso-López, Clara Martín-Martín, Guadalupe Tena-García, Inmaculada Casas, Francisco Pozo, Ana Méndez-Echevarría, Jara Hurtado-Gallego, Cristina Calvo

**Affiliations:** 1 Pediatrics Department, Severo Ochoa University Hospital, 28911 Leganés, Spain; marialuz.hso@gmail.com (M.L.G.-G.); alonsolopezpatricia@gmail.com (P.A.-L.); claramartinmir@gmail.com (C.M.-M.); guape95@gmail.com (G.T.-G.); 2 Centro de Investigación Biomédica en Red en Enfermedades Infecciosas (CIBERINFEC), Instituto de Salud Carlos III, 28029 Madrid, Spain; amendezes@yahoo.es (A.M.-E.); jarahurtadogallego@gmail.com (J.H.-G.); 3 Puerta de Hierro Health Research Institute (IDIPHISA), 28222 Majadahonda, Spain; 4 Traslational Research Network in Pediatric Infectious Diseases (RITIP), 28046 Madrid, Spain; 5 La Paz University Hospital, 28046 Madrid, Spain; 6 La Paz Institute for Health Research (IdiPAZ), 28029 Madrid, Spain; 7 Universidad Autónoma Madrid (UAM), 28029 Madrid, Spain; 8 Respiratory Virus and Influenza Unit, National Center of Microbiology, 28222 Madrid, Spain; icasas@isciii.es (I.C.); pacopozo@isciii.es (F.P.); 9 Centro de Investigación Biomédica en Red en Epidemiología y Salud Pública (CIBERESP), Instituto de Salud Carlos III, 28029 Madrid, Spain

**Keywords:** antibiotics, bronchiolitis, respiratory virus, respiratory syncytial virus, human bocavirus, human adenovirus, rhinovirus, chest X-ray

## Abstract

Bronchiolitis is a viral respiratory infection, with respiratory syncytial virus (RSV) being the most frequent agent, requiring hospitalization in 1% of affected children. However, there continues to be a noteworthy incidence of antibiotic prescription in this setting, further exacerbating the global issue of antibiotic resistance. This study, conducted at Severo Ochoa Hospital in Madrid, Spain, focused on antibiotic usage in children under 2 years of age who were hospitalized for bronchiolitis between 2004 and 2022. In that time, 5438 children were admitted with acute respiratory infection, and 1715 infants (31.5%) with acute bronchiolitis were included. In total, 1470 (87%) had a positive viral identification (66% RSV, 32% HRV). Initially, antibiotics were prescribed to 13.4% of infants, but this percentage decreased to 7% during the COVID-19 pandemic thanks to adherence to guidelines and the implementation of rapid and precise viral diagnostic methods in the hospital. HBoV- and HAdV-infected children and those with viral coinfections were more likely to receive antibiotics in the univariate analysis. A multivariate logistic regression analysis revealed a statistically independent association between antibiotic prescription and fever > 38 °C (*p* < 0.001), abnormal chest-X ray (*p* < 0.001), ICU admission (*p* = 0.015), and serum CRP (*p* < 0.001). In conclusion, following guidelines and the availability of rapid and reliable viral diagnostic methods dramatically reduces the unnecessary use of antibiotics in infants with severe bronchiolitis.

## 1. Introduction

Bronchiolitis is traditionally defined as the initial episode of expiratory dyspnea accompanied by catarrhal symptoms occurring within the first two years of life (McConnochie criteria) [1]. Through active surveillance, the presence of at least one virus can be identified in approximately 85% of cases, with 25% of cases exhibiting multiple viral infections [2]. Respiratory syncytial virus (RSV) accounts for 65–75% of the detected viruses, with even higher rates during traditional winter epidemics. The presence of coinfections, particularly when RSV is involved, might contribute to greater clinical severity, although consensus on this matter remains elusive within the scientific literature [3]. Among children under the age of two who develop bronchiolitis, approximately 1% necessitate hospitalization, with a notable proportion requiring respiratory support and intensive care. The peak incidence of hospitalization is observed between 3 and 6 months of age [4,5]. In Spain, the cumulative incidence of hospital admissions for bronchiolitis, as evidenced by data from a cohort of children under 24 months, ranges from 1% to 3.5% (with a rate of 5% among those under 3 months of age, 3.7% among those under 6 months, and 2.5% among those under 12 months) [6]. While certain established risk factors such as prematurity, age under 3 months, or comorbidities may increase the likelihood of a more severe outcome, the majority of hospitalized children are healthy infants.

The second most common virus is rhinovirus (HRV), responsible for approximately 15% of bronchiolitis cases [7]. HRV tends to be prevalent during the early autumn and spring seasons, although it can manifest at any time of the year. To a lesser extent, other viruses such as human bocavirus (HBoV), which typically circulates in conjunction with RSV during the winter and often presents as a co-infection, and human metapneumovirus (HMPV), which usually exhibits epidemics during the spring, also serve as causative agents of bronchiolitis. Human adenovirus (HAdV) has been frequently identified in co-infections as well [8]. In the case of SARS-CoV-2, it has been shown to induce bronchiolitis, albeit rarely, and generally results in milder symptoms in comparison to other viruses, such as RSV or even HRV [9,10].

Although it is a viral process, a high percentage of children with bronchiolitis receive treatment with antibiotics. Antibiotics have allowed great progress in the treatment of infectious diseases, reducing the morbidity and mortality of the population [11], but they have also been one of the great problems of humanity. According to the World Health Organization (WHO), bacterial resistance represents a significant threat to global public health because it reduces the effectiveness of medical treatments and can lead to more serious and difficult-to-treat infections. We are observing a spread of multi-resistant bacteria, and reducing antibiotic resistance is one of the main objectives of the WHO. It is estimated that antibiotic resistance is responsible for 700,000 deaths per year worldwide and 25,000 deaths per year in the EU. In addition, it could cause more mortality than cancer in the year 2050 [12,13].

Antibiotic resistance is a growing problem, and it is largely associated with excessive use of antibiotics, inadequate treatments, and a lack of awareness about the problem [13,14]

The pediatric population is one of the patient groups that receives the greatest number of antibiotic prescriptions [15] and is not immune to this problem. The fight against antibiotic resistance is today a global priority. It is essential to ensure that antibiotics are used rationally and only in those cases in which they are necessary.

This study aims to investigate the utilization of antibiotics in a cohort of children hospitalized for bronchiolitis and identify associated clinical and epidemiological characteristics.

## 2. Materials and Methods

This is a sub-study of an ongoing prospective investigation of respiratory tract infections in children, funded by grants from the Spanish Health Research Fund (FIS) under the reference numbers PI98/0310, PI06/0532, PI12/0129, PI15CIII/00028, PI18CIII/00009, PI21CIII/00019, and PI21/00377 and approved by the Medical Ethics Committee at University Hospital Severo Ochoa.

### 2.1. Study Population

The study population encompassed all children under the age of 2 diagnosed with acute bronchiolitis and admitted to the secondary public hospital Severo Ochoa in Leganés, Madrid, between September 2004 and August 2022. Informed consent was duly obtained from parents or legal guardians.

The Severo Ochoa Hospital is the only hospital in the city of Leganés with a population of 186,000, including approximately 32,000 children under the age of 14. The hospital lacks a pediatric intensive care unit (PICU), necessitating the transfer of children requiring such specialized care to a tertiary hospital. Subsequently, patients return to Severo Ochoa Hospital following their stay in the PICU.

Bronchiolitis was defined as the first episode of lower airway respiratory distress in children under 2 years of age [1]. All other episodes of acute expiratory wheezing were considered to be recurrent wheezing and were not included. Exclusion criteria were a refusal to participate in the study. 

### 2.2. Study Procedures

All patients were evaluated by an attending physician. Clinical and epidemiological characteristics of the patients were collected. During the hospital stay, and as part of the study, a physician filled out a study questionnaire with the following variables: age, sex, month of admission, clinical diagnosis, history of prematurity and underlying chronic diseases, need for oxygen therapy (evaluated via transcutaneous oxygen saturation), fever and maximum axillary temperature, presence of infiltrates and/or atelectasis in chest X-rays, administration of antibiotic therapy at any time during the admission, length of hospital stay, total white blood cell (WBC) count, C-reactive protein (CRP) serum levels and blood culture results (for those cases in which such tests had been performed), and the results of a virological study.

Specimens consisted of nasopharyngeal aspirates (NPAs) that were taken from each patient at admission. 

Antibiotic prescription was considered to be adequate when the patient was diagnosed with a bacterial infection in addition to bronchiolitis, or when the blood culture was positive. Urinary infections confirmed via a urine culture or concomitant acute otitis media, evaluated by a pediatrician and with redness and bulging of the eardrum or drainage, were considered bacterial infections.

### 2.3. Virological Study

NPAs were sent for virological investigation to the Influenza and Respiratory Viruses Laboratory at the National Center for Microbiology (ISCIII), Madrid, Spain. Samples were stored at 4 degrees Celsius in the refrigerator and processed within 24 h after collection. Upon reception, three aliquots were prepared and stored at −80 °C. 

Both the reception and the NPA-sample-processing areas were separated from those defined as working areas. RNA and DNA from 200 L aliquots of NPA were extracted using the QIAamp MinElute Virus Spin Kit in an automated extractor (QIAcube, Qiagen, Valencia, Spain). Respiratory virus detection was performed by four independent real-time multiplex PCR (RT-PCR) assays using the SuperScript III Platinum One-Step Quantitative RT-PCR System (Invitrogen^®^, Waltham, MA, USA). The first assay detected Influenza A, B, and C viruses; the second assay detected parainfluenza viruses 1 to 4 (PIV), hRV, and enteroviruses; the third assay detected RSV types A and B, human metapneumovirus (hMPV), human bocavirus (hBoV), and AdV. Human coronavirus (HCoV) was investigated using a generic RT-PCR that was able to detect human alpha and beta coronavirus, HCoV 229E/HCoV NL63, and HCoV OC43/HCoV HKU1. The primers and Taqman probes used in this study have already been reported by the study investigators [16]. In addition, the detection of SARS-CoV-2 was performed on an extracted RNA from NPAs from 2020 using a real-time RT-PCR assay based on the method designed by Corman et al. [17] for the specific amplification of the E gene using the One-Step RT-PCR Kit (NZYTech, Lisbon, Portugal). This method was adapted to our laboratory, including the amplification of an internal control from the sample in a multiplex way. Assay sensitivity was regularly assessed to check for potential failures in specificity associated with viral variability. Quality controls organized by the ECDC/WHO and QCMD were conducted annually to check the sensitivity and specificity of all of the tests used. The results of the virological study were available in 5–7 working days. After the onset of the COVID-19 pandemic, in addition to the virological study conducted for this research at the ISCIII, respiratory virus PCR testing was implemented in our hospital, with results available within the day.

### 2.4. Statistical Analysis 

Descriptive data were expressed as means and standard deviations (SDs) for continuous variables and through counts and percentages for categorical variables. Continuous variables that followed a normal distribution were compared using a one-way analysis of variance with a Bonferroni correction or through *t*-tests. Categorical variables were compared using the chi-squared test or Fisher’s exact test, as appropriate. 

Logistic regression models were constructed to evaluate a range of potential risk factors associated with antibiotic prescription. Each variable was individually introduced into univariate models, and odds ratios (ORs) with corresponding 95% confidence intervals (CIs) were computed. Explanatory factors with *p*-values  <  0.1 in the univariate analysis were subsequently analyzed in a multiple regression model. A multivariate backward stepwise logistic regression model was employed to determine adjusted ORs along with 95% CIs, enabling the estimation of independent associations between various factors and antibiotic prescription. 

A *p*-value of less than 0.05 was considered statistically significant. All analyses were two-tailed and were performed using the Statistical Package for the Social Sciences (SPSS), version 25; SPSS Inc., Chicago, IL, USA. 

## 3. Results

A total of 5438 children were admitted to Severo Ochoa Hospital with acute respiratory infections between September 2004 and August 2022. Of these, 1715 infants under the age of 2, who were diagnosed with acute bronchiolitis, consented to participate in the study. The average age of the participants was 5.5 ± 6.7 months, with males comprising 57% of the cohort. Table 1 provides a summary of the primary clinical and demographic characteristics of the study group.

### 3.1. Respiratory Virus Findings

At least one respiratory virus was detected in 1490 (87%) cases. The most commonly identified virus was RSV, found in 977 cases (66%), followed by HRV in 480 (32%), HAdV in 128 (9%), HBoV in 98 (6.6%), and HMPV in 90 (6%). Viral coinfections were detected in 453 cases, representing 26% of positive cases, with the most common coinfection being RSV and HRV in 151 cases, accounting for 33% of all coinfections. 

### 3.2. Factors Associated with Antibiotic Prescription during Hospitalization

Overall, antibiotic treatment was prescribed to 229 (13.4%) infants, showing a notable decline from 2020 onwards, aligning with the onset of the COVID-19 pandemic, dropping from 14% before 2020 to 7% thereafter (*p* = 0.038; OR: 2.1; 95% CI: 1.1–4.1). Of the children treated with an antibiotic, 41 (18%) were diagnosed with acute otitis media and 11 (5%) with urinary tract infection. A bacterial pathogen was detected in a blood culture only in two (0.1%) patients: *Streptococcus pneumoniae* (1) and *Moraxella catharralis* (1).

A higher proportion of children with HBoV (27.6%) and HAdV (25%) infections received antibiotics in comparison to those with RSV (13.7%), HRV (14%), HMPV (9%), influenza (10%), and PIV (8%) in the univariate analysis. Additionally, patients with viral coinfections were 1.6 times more likely to be treated with antibiotics than those with single infections (17.9% vs. 11.7%, *p* = 0.001).

Infants who were prescribed antibiotics were more likely to be older than 6 months (*p* < 0.001), exhibit symptoms such as fever (*p* < 0.001), experience hypoxia (*p* < 0.001), and require admission to the ICU (*p* < 0.001). Furthermore, a longer duration of fever (*p* < 0.001) and hypoxia (*p* = 0.001) were significantly associated with antibiotic prescription. Patients who received antibiotic treatment also had a notably prolonged hospital stay (Table 2).

Patients with abnormal chest X-rays (infiltrate/atelectasis) were six times more likely to receive antibiotic prescriptions (*p* < 0.001). Notably, there was a remarkable decrease in the request for chest X-rays throughout the study period. Starting in 2015, following the publication of the 2014 AAP guideline for bronchiolitis management, the percentage of chest X-ray requests decreased from 74% to 45% (*p* < 0.001). The factors associated with a request for chest X-rays are detailed in Table 3. 

After a multivariate logistic regression analysis, the factors independently associated with the performance of a chest X-ray were age greater than 6 months (*p* = 0.04, OR: 2.6, 95% CI: 1.1–6.6), duration of fever, and hypoxia (*p* = 0.07 and *p* = 0.03, respectively), and admission prior to the publication of the 2014 AAP guideline (*p* = 0.001, OR: 4.1, 95% CI: 1.7–9.6). Significantly elevated levels of both serum CRP (*p* < 0.001) and white blood cell count (*p* = 0.008) were observed in the group of patients treated with antibiotics compared to those who were not treated.

Patients with fever (N = 866) were examined, and the findings are presented in Table 4. Among them, 191 (22%) were administered antibiotics. This subgroup exhibited significantly prolonged durations of fever, hypoxia, and hospital admission. They displayed infiltrates on X-rays and required admission to the PICU more frequently. Although their CRP levels were elevated, there was no significant increase in leukocytosis. Furthermore, an analysis was conducted on children admitted to the PICU, comparing those who received antibiotics to those who did not (Table 5). Children who received antibiotics experienced more frequent fevers and had longer hospital stays. Elevated CRP levels were also observed in this group. However, there were no significant differences in radiological infiltrates or the need for mechanical ventilation between children with and without antibiotic therapy.

We conducted a comparative analysis of antibiotic usage before and after the implementation of the 2014 AAP guideline. Although there was a decrease in antibiotic use following the AAP guideline implementation, with a rate of 21.9% (396 cases) in contrast to the 23.9% (967 cases) observed prior to the guideline, this difference did not reach statistical significance (*p* = 0.091, OR 1.12 (95% CI: 0.98–1.29)). Similarly, a comparison of antibiotic utilization before and after the implementation of multiplex respiratory PCR in our hospital did not reveal statistically significant changes. The rates were 23.4% (1034 cases) before PCR implementation and 22.4% (229 cases) after PCR implementation (*p* = 0.492, OR 1.06 (95% CI: 0.9–1.24)). Additionally, we assessed the duration of hospitalization before and after the introduction of multiplex respiratory PCR in our hospital and found that the hospital stay was longer during the period preceding the rapid PCR diagnosis implementation, with a mean and standard deviation of 4.01 (2.72) compared to 3.57 (2.32), *p* < 0.001.

No differences regarding passive smoking, family history of atopy, or breastfeeding were found. 

A multivariate logistic regression analysis revealed a statistically independent association between antibiotic prescription and the following variables: fever > 38 °C, *p* < 0.001, abnormal chest-X ray, *p* < 0.001, PICU admission, *p* = 0.015, and serum C reactive protein, *p* < 0.001. See Table 6 for details. Patients with HBoV infections showed a tendency to receive antibiotics more frequently, although without reaching statistical significance (*p* = 0.07).

## 4. Discussion

In this study conducted on a prospective cohort of more than 1700 infants hospitalized for bronchiolitis between 2004 and 2022 in Spain, we observed that 13.4% of them received antibiotic treatment despite a lack of evidence of bacterial infection. This percentage, while lower than that reported in most studies, could likely be further reduced. Antibiotic prescription was considered appropriate in our series in 22.7% of cases in which a bacterial infection was diagnosed. The main independent risk factor associated with antibiotic prescription was the presence of infiltrates or atelectasis in the chest X-ray, leading to a fivefold increase in the probability. Additionally, other factors independently associated with antibiotic treatment included the presence of fever, admission to the PICU, elevated serum CRP, and HBoV infection. 

In numerous guidelines, it is recommended not to prescribe antibiotic treatment to children diagnosed with bronchiolitis unless there is evidence of bacterial superinfection [18,19]. However, it still prevails that these patients, particularly those with a more severe condition, are prescribed antibiotics, potentially influenced by the perception of severity among prescribers. Some studies, including one conducted by Obolski in Israel [20], reported an antibiotic therapy rate of up to 33% among children hospitalized for bronchiolitis. Consistent with our findings, antibiotics were prescribed more frequently to children with fever and more severe symptoms, as well as in those with previous visits to the emergency room. In the USA, between 2007 and 2015, up to a quarter of children with bronchiolitis received antibiotic treatment [21], and this proportion was even higher in Italy, reaching half of all infants [22].

Nonetheless, bacterial coinfections are infrequent in children with bronchiolitis, occurring in approximately 1–2% of cases, including urinary infections. The incidence increases in patients requiring mechanical ventilation [23,24], but it remained uncommon in our series and generally in bronchiolitis cases. Among our patients treated with antibiotics, only 0.1% had a positive blood culture, and 5% had a positive urinary culture.

It is widely acknowledged that certain viruses, such as HAdV and HBoV, are associated with high fever and a significant increase in C-reactive protein levels, which may mimic bacterial infections. Both viruses are also frequently associated with infiltrates on chest X-rays [8,25,26]. The combination of these factors may justify the suspicion of bacterial infection and lead to increased antibiotic prescriptions, as observed in our series, especially in cases of HBoV infections. In a previous study by our research team involving 319 HBoV infections, we observed that 68% of the cases had fever, 47% had an infiltrate on X-ray, and CRP levels were moderately elevated (25,26). Identifying the etiology of the episode, as is often the case with RSV infections, thanks to the routine availability of rapid tests in emergency departments, can serve as a valid rationale for reducing antibiotic prescriptions. 

Regarding the value of CRP, it is somewhat controversial in the literature [27]. Procalcitonin (PCT) and CRP are non-specific markers of the host response to tissue injury and inflammation, and their serum concentrations usually are higher in bacterial than in viral respiratory tract infections. Although PCT has shown somewhat better performance in detecting bacterial infections, especially in children with bronchiolitis in the PICU [28], neither of the two markers are usually in high ranges in viral infections [28]. It is considered that CRP values above 80–100 mg/L are associated with bacterial superinfections, but these figures are rarely reached in children with bronchiolitis, and many viral infections moderately elevate CRP overall. Alejandre et al., upon assessing 706 Spanish infants treated in the PICU for bronchiolitis, consider that there is no need to treat an invasive bacterial infection as long as PCT stays at the level of <1.0 ng/mL and CRP < 70 mg/L. In our study, the mean CRP is generally below this figure, even in patients admitted to the PICU. Unfortunately, PCT was not used throughout the entire period and could not be analyzed. Possibly, the use of markers such as PCT or CRP should be accompanied by the implementation of clinical practice guidelines to translate well into the management of antibiotics in children with bronchiolitis [29].

In our study, there was a decrease in antibiotic usage over time, possibly influenced by two factors. Firstly, there was a reduction in the number of radiographs requested between the earlier years and the more recent ones. It is recognized that viral infections, including RSV, which is the most common virus, give rise to radiological infiltrates, atelectasis, and viral pneumonia. Although they can probably increase the severity of the condition, they do not require antibiotic treatment, but performing a chest X-ray may induce more antibiotic prescriptions. In this regard, better adherence to guidelines has been described as a factor that prevents antibiotic therapy [30]. In addition to the international guidelines, local guidelines were also published in Spain that have possibly influenced this reduction in chest X-ray performance [31]. The training of physicians and the implementation of antibiotic stewardship programs are also crucial factors to consider in reducing inappropriate prescriptions [32,33]. 

Secondly, and perhaps more importantly, it is worth noting that in the pre-pandemic era, the virological diagnosis of respiratory infections in our hospital was conducted through the ISCIII as part of this study, with a delay in obtaining results of nearly a week. However, with the onset of the pandemic, the hospital implemented local viral diagnosis via PCR for respiratory viruses. This strategy allowed for much quicker access to virological results, enabling therapeutic decisions to be made in accordance with them and resulting in a 50% reduction in antibiotic prescriptions in children admitted with bronchiolitis. This outcome underscores the significant importance of implementing molecular diagnostic techniques in hospitals as, as in our case, it can lead to a 50% reduction in unnecessary antibiotic prescriptions.

The inappropriate use of antibiotics fosters the development of antimicrobial resistance, can contribute to the onset of allergies, and disrupts the intestinal and respiratory microbiota during a critical period when a child’s immune system is maturing [34]. For all these reasons, it is crucial to carefully select the children diagnosed with bronchiolitis who truly require this treatment. 

Thorough adherence to clinical practice guidelines, the effective implementation of antimicrobial stewardship programs and the establishment of precise virological diagnoses collectively contribute to the judicious use of antibiotic treatments. The strict adherence to bronchiolitis management protocols has shown a substantial reduction in antibiotic use and an overall enhancement in treatment quality. Therefore, these initiatives should be extended to other healthcare settings [35]. Education on the correct use of antibiotics must reach not only prescribers but also parents and caregivers who must understand their children’s illness. Pressure from family members can be a factor that determines the use of antibiotics, and educational programs for the population are also necessary. At our hospital, we are working on the development of educational materials for parents and caregivers.

## 5. Conclusions

In this long-term study, it was observed that the prescription of antibiotic treatment to infants with bronchiolitis can be improved and reduced when virological diagnostic results are made available quickly and accurately. The key factors associated with antibiotic prescription included fever, chest X-ray infiltrates, and the need for PICU admission. 

The publication of treatment guidelines, the decrease in the number of radiographs performed, and the greater availability of virological etiological diagnosis could be behind the improvement in antibiotic prescription over time. The establishment of antibiotic stewardship and education programs should be a priority in this pathology.

## Figures and Tables

**Table 1:** Clinical characteristics of 1715 infants hospitalized with acute viral bronchiolitis.

Clinical Characteristic	N (%)
Age (months) *	5.5 ± 6.7
Male	984 (57%)
Prematurity	212 (12%)
Temperature > 38 °C	866 (51%)
Fever duration (days) *	2.7 ± 2.2
Highest temperature (°C) *	38.6 ± 0.5
Hypoxia (SatO_2_ < 95%)	1156 (67%)
Hypoxia duration (days) *	3.2 ± 2.5
Length hospital stay (days) *	4.5 ± 2.9
Abnormal chest radiograph	415 (24%)
Antibiotic treatment	229 (13%)
PICU admission	68 (4%)
Leucocytes (cells/mm^3^)	13,446 ± 9533
Serum C reactive protein (mg/L) *	26.6 ± 39.0

* mean ± standard deviation. PICU: pediatric intensive care unit.

**Table 2:** Clinical characteristics of infants admitted for bronchiolitis (N = 1715), treated or not with antibiotics.

Clinical Characteristics	Antibiotic Treatment N = 229	No Antibiotic Treatment N = 1486	*p*-Value	OR (95% CI)
Sex, male	120 (52%)	864 (58%)	0.102	0.8 (0.6 to 1.0)
Age (months) *	7.3 ± 5.9	5.2 ± 6.7	<0.001	
Age > 6 months	113 (49%)	437 (29%)	<0.001	2.3 (1.8 to 3.1)
Prematurity	29 (13%)	183 (12%)	0.881	1.0 (0.7 to 1.6)
Fever > 38 °C	191 (84%)	675 (45%)	<0.001	6.2 (4.3 to 8.9)
Duration of fever (days) *	3.3 ± 3.1	2.4 ± 1.9	<0.001	
Hypoxia	183 (80%)	973 (66%)	<0.001	2.1 (1.5 to 3.0)
Duration of hypoxia (days) *	3.8 ± 2.8	3.1 ± 2.4	0.001	
Length of hospital stay (days) *	6.3 ±3.4	4.4 ± 2.8	<0.001	
Infiltrate/atelectasis	150 (72%)	265 (29%)	<0.001	6.2 (4.4 to 8.7)
PICU admission	22 (10%)	46 (3%)	<0.001	3.1 (1.8 to 5.3)
Respiratory virus detection	197 (86%)	1293 (87%)	0.599	0.9 (0.6 to 1.4)
Viral coinfection	81 (36%)	372 (25%)	0.001	1.6 (1.2 to 2.2)
Respiratory syncytial virus infection	134 (59%)	843 (58%)	0.636	1.1 (0.8 to 1.4)
Rhinovirus infection	67 (30%)	413 (28%)	0.660	1.1 (0.8 to 1.5)
Adenovirus infection	32 (14%)	96 (7%)	<0.001	2.3 (1.5 to 3.6)
Human metapneumovirus infection	8 (4%)	82 (6%)	0.198	0.6 (0.3 to 1.3)
Human bocavirus infection	27 (12%)	71 (5%)	<0.001	2.7 (1.7 to 4.2)
Influenza infection	5 (2%)	45 (3%)	0.476	0.7 (0.3 to 1.8)
Parainfluenza virus infection	8 (4%)	93 (6%)	0.096	0.5 (0.3 to 1.1)
Leucocytes (cells/mm^3^) *	15,046 ± 9349	12,940 ± 9694	0.008	
Serum C reactive protein (mg/L) *	45.2 ± 52.6	20.1 ± 30.4	<0.001	

* mean ± standard deviation. PICU: pediatric intensive care unit.

**Table 3:** Factors associated with a request for chest X-rays in infants hospitalized for bronchiolitis.

Clinical Characteristics	Chest X-ray Performed N = 1109	Chest X-ray not Performed N = 605	*p*-Value	OR (95% CI)
Sex, male	610 (55%)	373 (62%)	0.006	0.8 (0.6 to 0.9)
Age (months) *	6.3 ± 7.5	4.0 ± 4.3	<0.001	
Age > 6 months	428 (39%)	121 (20%)	<0.001	2.5 (2.0 to 3.2)
Fever > 38 °C	679 (61%)	186 (31%)	<0.001	3.5 (2.9 to 4.4)
Duration of fever (days) *	2.9 ± 2.3	1.9 ± 1.5	<0.001	
Hypoxia	815 (74%)	338 (56%)	<0.001	2.1 (1.7 to 2.6)
Duration of hypoxia (days) *	3.5 ± 2.8	2.6 ± 1.7	0.001	
Length of hospital stay (days) *	5.2 ±3.2	3.6 ± 2.	<0.001	
PICU admission	62 (6%)	5 (0.9%)	< 0.001	6.8 (2.7 to 16.9)
Adenovirus infection	96 (9%)	32 (5%)	<0.001	2.3 (1.5 to 3.6)
Human bocavirus infection	82 (8%)	16 (3%)	<0.001	2.9 (1.7 to 5.1)
Parainfluenza virus infection	52 (5%)	49 (8%)	0.005	0.6 (0.4 to 0.8)
Leucocytes (cells/mm^3^) *	13,827 ± 10176	11,386 ± 4437	<0.001	
Serum C reactive protein (mg/L) *	28.7 ± 40.7	14.6 ± 19.5	<0.001	

* mean ± standard deviation. PICU: pediatric intensive care unit.

**Table 4:** Clinical characteristics of infants admitted for bronchiolitis with fever (N = 866), treated or not with antibiotics.

Clinical Characteristics	Antibiotic Treatment N = 191	No Antibiotic Treatment N = 675	*p*-Value	OR (95% CI)
Sex, male	106 (56%)	366 (54%)	0.755	1.1 (0.8 to 1.5)
Age (months) *	7.8 ± 5.9	6.5 ± 6.3	0.016	
Prematurity	19 (10%)	61 (9%)	0.701	1.1 (0.6 to 1.9)
Duration of fever (days) *	3.4 ± 3.1	2.4 ± 1.9	<0.001	
Hypoxia	153 (81%)	483 (72%)	0.015	1.6 (1.1 to 2.4)
Duration of hypoxia (days) *	3.8 ± 2.8	3.1 ± 2.3	0.001	
Length of hospital stay (days) *	6.1 ±3.4	4.7 ± 2.7	<0.001	
Infiltrate/atelectasis	132 (74%)	176 (35%)	<0.001	5.2 (3.5 to 7.6)
PICU admission	14 (7%)	17 (3%)	0.003	2.9 (1.4 to 6.0)
Respiratory virus detection	162 (86%)	597 (90%)	0.104	0.7 (0.4 to 1.1)
Leucocytes (cells/mm^3^) *	15,125 ± 9564	13,409 ± 11,265	0.086	
Serum C reactive protein (mg/L) *	49.1 ± 54	21.5 ± 22.3	<0.001	

* mean ± standard deviation. PICU: pediatric intensive care unit.

**Table 5:** Clinical characteristics of infants admitted to the Pediatric Intensive Care Unit (PICU) for bronchiolitis (N = 68) who were treated with antibiotics and those who were not.

Clinical Characteristics	Antibiotic Treatment N = 22	No Antibiotic Treatment N = 46	*p*-Value	OR (95% CI)
Sex, male	11 (50%)	26 (57%)	0.613	0.8 (0.3 to 2.1)
Age (months) *	4.1 ± 6.0	2.9 ± 4.0	0.316	
Prematurity	6 (27%)	9 (20%)	0.473	1.5 (0.5 to 5.1)
Fever > 38 °C	14 (67%)	17 (37%)	0.024	3.4 (1.2 to 10.1)
Duration of fever (days) *	2.4 ± 1.9	1.5 ± 0.8	0.074	
Duration of hypoxia (days) *	7.5 ± 4.4	5.4 ± 3.2	0.050	
Length of hospital stay (days) *	9.3 ± 5.7	7.4 ± 4.9	0.169	
Length of PICU stay (days) *	5.6 ± 4.2	2.2 ± 2.4	0.029	
Mechanic ventilation	3 (50%)	7 (54%)	0.876	0.9 (0.1 to 5.9)
Infiltrate/atelectasis	13 (65%)	17 (41%)	0.071	2.7 (0.9 to 8.3)
Leucocytes (cells/mm^3^) *	16,367 ± 8547	14,615 ± 11,865	0.560	
Serum C reactive protein (mg/L) *	57.1 ± 50.1	14.8 ± 17.7	<0.001	

* mean ± standard deviation. PICU: pediatric intensive care unit.

**Table 6:** Multivariate analysis of factors associated with antibiotic prescription in infants admitted for bronchiolitis.

Clinical Characteristic	*p*-Value	Adjusted OR 95% CI
Temperature > 38 °C	<0.001	3.1 (1.8 to 5.3)
Infiltrate/atelectasis	<0.001	5.0 (3.2 to 7.6)
PICU admission	0.015	2.5 (1.2 to 5.3)
Serum C reactive protein (mg/L) *	<0.001	

* mean ± standard deviation. PICU: pediatric intensive care unit.

## Data Availability

The data presented in this study are available upon request from the corresponding author.
